# Supplementary material for: Epidemiological Characteristics and Factors Associated with Alzheimer’s Disease and Mild Cognitive Impairment among the Elderly in Urban and Rural Areas of Hubei Province
Source: J Clin Med. 2022 Dec 20;12(1):28. doi: 10.3390/jcm12010028 (PMC9820890; doi:10.3390/jcm12010028)
Supplement: Supplementary file 1 [file jcm-12-00028-s001.zip › jcm-2025364-supplementary.pdf]

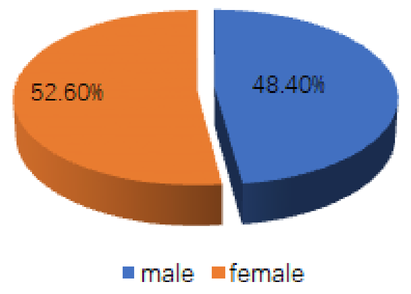

Figure S1. Gender composition of the sample

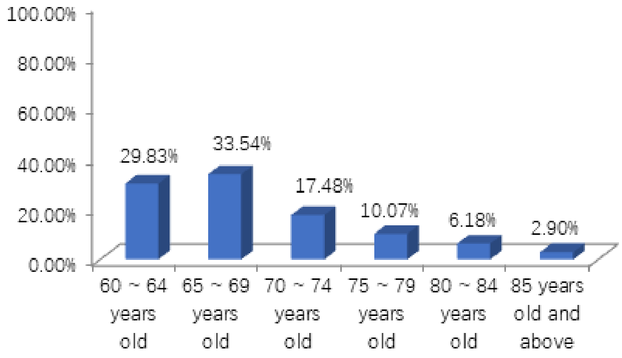

Figure S2. Age composition of the sample

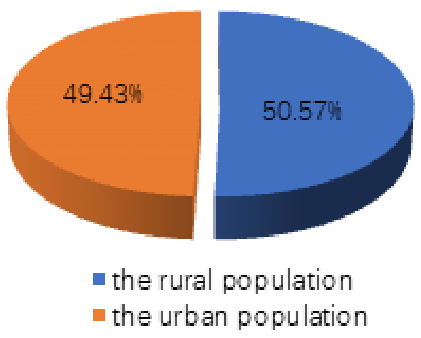

Figure S3. Regional composition of the sample

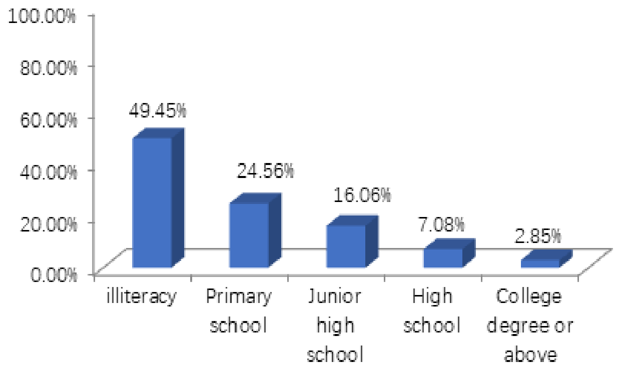

Figure S4. Education level of the sample

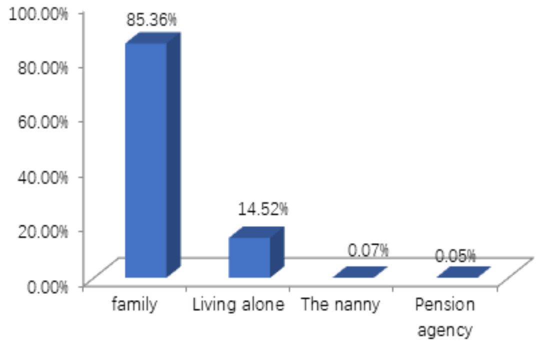

Figure S5. Residential situation of the sample

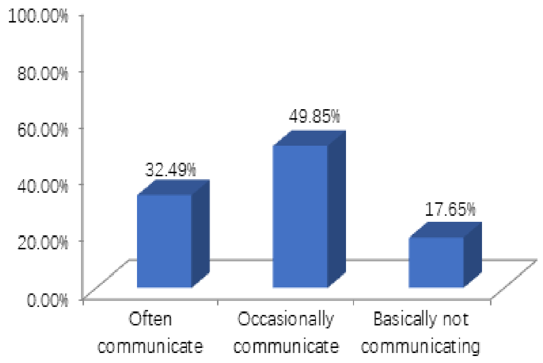

Figure S6. Communication between the participants and their children

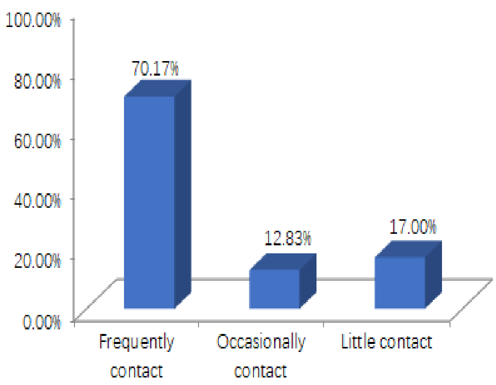

Figure S7. The interaction between the participants and their neighbours

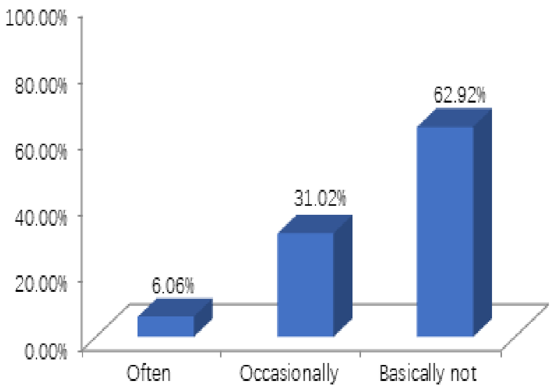

Figure S8. Participants' participation in social activities

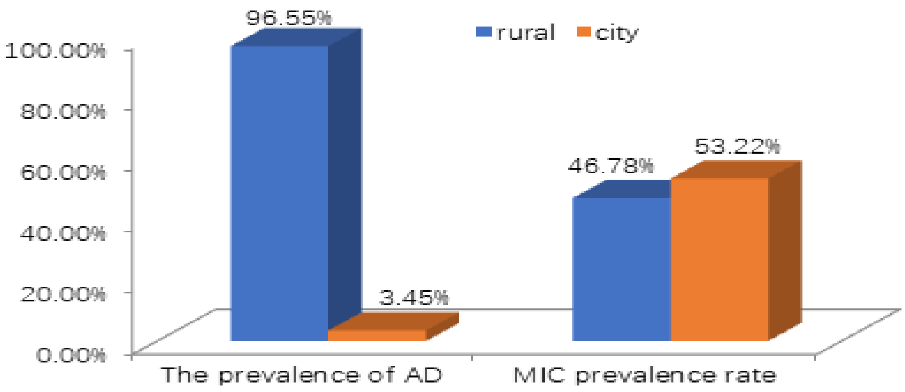

Figure S9. The proportion of patients with AD and MCI in different regions

Table S1. MMSE scores of AD patients

MMSE (points)	Cases (*n*)	The proportion (%)
0~3	5	8.6
4~6	6	10.3
7~9	16	27.6
10~12	14	24.1
13~15	7	12.1
16~18	10	17.2
Total	58	100

Table S2. MMSE scores of MCI patients

MMSE (points)	Cases (*n*)	The proportion (%)
1~11	50	12.38
12~14	41	10.15
15~17	49	12.13
18~20	77	19.06
21~23	89	22.03
24~26	98	24.26
Total	404	100

Table S3. Influence of social connections and life events on AD

Variable	*β*	*sx*	χ^2	*P*	*OR*（*95% CI*）
Children Communication	−0.344	0.235	2.144	0.143	0.71 [0.45,1.12]
Neighbour interaction	−0.376	0.179	4.435	0.035	0.69 [0.48,0.97]
Social activities	0.000	0.259	0.000	1.000	1.00 [0.60,1.66]
Working conditions	−0.105	0.324	0.105	0.746	0.90 [0.48,1.70]
Living situation	−0.483	0.344	1.979	0.159	0.62 [0.31,1.21]

Table S4. Influence of social connections and life events on MCI

Variable	*β*	*sx*	χ^2	*P*	*OR*（*95% CI*）
Children Communication	−0.276	0.092	8.974	0.003	0.76 [0.63,0.91]
Neighbour interaction	0.303	0.113	7.165	0.007	1.35[1.09,1.70]
Social activities	−0.257	0.118	4.749	0.029	0.77 [0.61,0.97]
Working conditions	0.147	0.151	0.953	0.329	1.16 [0.86,1.56]
Living situation	−0.167	0.205	0.665	0.415	0.85 [0.57,1.26]

Table S5. Influence of lifestyle behaviours on AD

Variable	*β*	*sx*	χ^2	*P*	*OR*（*95% CI*）
Regular check-up	−0.676	0.308	4.819	0.028	0.51 [0.28,0.93]
Smoking	0.441	0.204	4.675	0.031	1.55 [1.04,2.32]
A cup of tea	−1.667	0.771	4.684	0.030	0.19 [0.04,0.85]
Drinking	−0.237	0.314	0.569	0.451	0.79 [0.43,1.46]
Physical activity	0.081	0.229	0.127	0.722	1.09 [0.69,1.70]

Table S6. Influence of lifestyle behaviours on MCI.

Variable	*β*	*sx*	χ^2	*P*	*OR*（*95% CI*）
Sleep	0.791	0.152	27.147	0.001	2.21 [1.64,2.97]
Drinking	0.299	0.124	5.841	0.016	1.35 [1.06,1.72]
Physical activity	−0.115	0.098	1.373	0.241	0.89 [0.74,1.08]
A cup of tea	0.185	0.184	1.014	0.314	1.20 [0.84,1.73]
Smoking	0.036	0.12	0.09	0.764	1.04 [0.82,1.31]

Table S7. Influence of somatic diseases on AD

Variable	*β*	*sx*	χ^2	*P*	*OR*（*95% CI*）
Hypertension	0.060	0.315	0.036	0.850	1.06 [0.57,1.97]
Diabetes	−0.948	1.061	0.798	0.372	0.39 [0.05,3.10]
Coronary heart disease (CHD)	0.119	0.537	0.049	0.825	1.13 [0.39,3.23]
Stroke	−3.595	2.911	1.525	0.217	0.03 [0.00,8.25]
Chronic bronchitis	0.249	0.484	0.265	0.607	1.28 [0.50,3.31]
Asthma	0.693	1.225	0.320	0.571	2.00 [0.18,22.07]

Tuberculosis (TB)	0.000	1.179	0.000	1.000	1.00 [0.10,10.07]
Arthritis	0.301	0.684	0.194	0.659	1.35 [0.35,5.17]
Cervical spondylosis	0.089	0.469	0.036	0.849	1.09 [0.44,2.74]
Disc herniation	−1.654	1.040	2.528	0.112	0.19 [0.03,1.47]
Deaf	−3.346	3.584	0.872	0.350	0.04 [0.00,39.55]
The tumor	−3.346	4.389	0.581	0.446	0.04 [0.00,191.75]

Table S8. Influence of somatic diseases on MCI

**Variable**	***β***	***sx***	χ^2	***P***	***OR*（*95% CI*）**
Coronary heart disease (CHD)	0.819	0.235	12.118	0.001	2.27 [1.43,3.60]
Stroke	0.442	0.247	3.208	0.073	1.56 [0.96,2.52]

Table S9. Influence of hobbies on AD

**Variable**	***β***	***sx***	χ^2	***P***	***OR*（*95% CI*）**
Reading	−1.761	0.911	3.736	0.053	0.17 [0.03,1.03]
Chess	−1.882	0.896	4.408	0.036	0.15 [0.03,0.88]
Surf the Internet	−1.766	2.488	0.504	0.478	0.17 [0.00,22.43]

Table S10. Influence of hobbies on MCI

**Variable**	***β***	***sx***	χ^2	***P***	***OR*（*95% CI*）**
Reading	−0.738	0.259	8.097	0.004	0.48 [0.29,0.80]
Chess	−0.318	0.19	2.818	0.093	0.73 [0.50,1.06]
Surf the Internet	−0.212	0.199	1.137	0.286	0.81 [0.55,1.20]
